# Paradigm-HF: a Paradigm Shift in Heart Failure Treatment?

**DOI:** 10.5935/abc.20160009

**Published:** 2016-01

**Authors:** Luis Cláudio Lemos Correia, Anis Rassi Jr.

**Affiliations:** 1 Escola Bahiana de Medicina e Saúde Pública, Salvador, BA - Brazil; 2 Hospital São Rafael, Salvador, BA - Brazil; 3 Anis Rassi Hospital, Goiânia, GO - Brazil

**Keywords:** Heart Failure/treatment, Neprilysin/therapeutic use, Angiotensin-Converting Enzyme Inhibitors / therapeutic use.

In the first semester of this year, the U.S. Food and Drug Administration (FDA) approved a new drug for the treatment of heart failure, LCZ696, commercially known as *Entresto*. This new treatment option was evaluated by the FDA on a priority basis (fast track designation), which allowed a faster release than usual. In Brazil, the drug is being evaluated by the National Health Surveillance Agency (Agência Nacional de Vigilância Sanitária- ANVISA), similarly to what occurs in Canada and the European Union countries.

The scientific evidence that has supported the approval of the new drug by the FDA was primarily obtained from the results of the PARADIGM-HF (Prospective comparison of ARNI with ACEI to Determine Impact on Global Mortality and morbidity in Heart Failure) randomized clinical trial,^1^ which proved that LCZ696 was more effective than enalapril in a sample of more than 8,000 patients with systolic heart failure.

The enthusiasm for the new drug is due to the fact that this is the first, among several drugs tested over more than 20 years, to demonstrate increased efficacy when compared to traditional treatment with Angiotensin-Converting Enzyme (ACE) inhibitors, beta-blockers and spironolactone. However, in the presence of exaggerated enthusiasm, one must carefully evaluate how much this feeling is proportional to the level of evidence.

In a systematic analysis, we can say that the PARADIGM-HF trial has a low risk of bias and random errors when concluding that the LCZ696 is superior to enalapril at a dose of 20 mg daily. As for the size of the benefit, 21 patients need to use LCZ696 rather than enalapril for 27 months to prevent an event (death from cardiovascular causes or hospitalization for heart failure). This effect is qualitatively and quantitatively relevant. But does the PARADIGM-HF, in fact, represent a change in the paradigm of heart failure treatment?

## The tested concept

Although LCZ696 appears to be the name of a newly invented molecule, it is not exactly that. In fact, it is a mixture of 320 mg of traditional valsartan with sacubitril. Sacubitril is the new drug, which acts by inhibiting neprilysin action. Neprilysin degrades "good" molecules, such as the natriuretic peptide and bradykinin. Therefore, by inhibiting neprilysin, sacubitril increases the concentration of these molecules, which have vasodilating and natriuretic action. Therefore, the burden of proof is on the clinical benefit of sacubitril. Surprisingly, this was not the concept tested in PARADIGM-HF trial.

What has been done in the PARADIGM-HF trial? The standard treatment of the sacubitril group was curiously more appropriate than the standard treatment in the control group. While the sacubitril group patients enjoyed a blockade of the renin-angiotensin-aldosterone system determined by a maximum dose of valsartan (320 mg daily), in the control group patients were given half of the maximum dose of enalapril (20 mg daily, fixed dose).

The correct method and commonly used in clinical trials to test the efficacy of a new therapeutic strategy is to randomize patients to a new treatment vs. placebo, making the standard treatment similar between the two groups, through the mere effect of randomness. One would therefore randomize patients to sacubitril vs. placebo, without interfering with the adjunctive treatment. In this case, sacubitril itself would be tested, not an association represented by the curious name of LCZ696. Hence, the baseline treatment received by the patients would not represent a confounding factor.

### Insufficient justification

The authors affirm in their article that simultaneous inhibition of ACE and of neprilysin should be avoided due to the risk of angioedema, justifying avoiding an association of sacubitril with enalapril in the study protocol. The references used to generate such concern come from studies with omapatrilat, a drug that inhibits these two systems and has been associated with 0.8% angioedema, compared to 0.5% angioedema in the control group.^2^

Regardless of the weakness of this argumentation, if the intention was to prevent such an association, there would be an alternative to avoid the confounding factor of heterogeneous adjunctive treatment between the groups: randomize patients to sacubitril and valsartan vs. placebo and valsartan, at the same dose. Thus, the groups would receive the same treatment, with the only difference being represented by the presence of sacubitril.

It can be observed that neither of the two alternatives to avoid the confounding effect was used in the study design, making it impossible to know which concept was actually tested. Was the higher efficacy of LCZ696 due to the advent of sacubitril or greater blockage of the renin-angiotensin-aldosterone system?

It was not only different adjunct drugs that were used in both groups. The enalapril dose was proportionally lower than the valsartan dose. The study authors justify the enalapril dose in the PARADIGM-HF trial by stating that this is the mean dose of the major studies that validated the efficacy of this drug, the SO LVD and the CONSENSUS (Cooperative North Scandinavian Enalapril Survival Study) trial.^3,4^ The dose of 40 mg enalapril was proposed in CONSENSUS trial, although a minority reached this dose, with a mean dose of 17 mg. The PARADIGM-HF study patients were functional class II/III, with SOLVD being more representative of that population. In the SOLVD trial, the target dose ceased to be 40 mg to be 20 mg, thus equal to that of PARADIGM-HF.

The affirmation that the enalapril dose in PARADIGM-HF is similar to that used in these studies has been naively accepted by some as a justification for the methodology.^5^ However, this does not free the study from the potential confounding effect represented by the methodology. Regardless of the "clinical logic" when choosing the working method, the adjunctive sacubitril treatment was significantly different between the groups. There is a great difference between choosing a medication dose to treat an individual patient and a study protocol that aims to demonstrate a concept of efficacy. In the latter, there must be concern for confounding bias.

Furthermore, the fixed dose of 20 mg a day of enalapril used in the study is not the same as a mean dose of 20 mg a day, which results from the individualization according to the patient, using higher doses in some and lower doses in others. A mean dose of 20 mg a day is probably more effective than a fixed dose of 20 mg a day.

Therefore, one cannot state with certainty that sacubitril represents the long-awaited evolution in the treatment of heart failure. The study is vulnerable to the confounding effect generated by an inadequate method to test the concept of efficacy of the drug active principle.

### The run-in phase

There is a second problem with the PARADIGM-HF trial, related to the demonstration of tolerability and safety of the LCZ696 scheme. This study presents an underutilized strategy in phase III-clinical trials: a run-in phase. Before being randomized, patients were submitted, in an open manner, for 4 to 6 weeks of treatment with LCZ696 and only those who tolerated the drug were included in the study. Thus, the study is related only to patients who are tolerant (initially) to LCZ696, which reduces its external validity regarding safety outcomes. If someone decides to replace the old ACE inhibitor by LCZ696, they should know that there is a greater probability of intolerance in their patient, compared to that observed in the study.

## Conclusion

From our point of view, rather than assessing the issue from the perspective of a fast track, regulatory agencies should, unhurriedly, question the industry why they chose a design that does not adequately evaluate the effectiveness of the new molecule, using a less effective blockade of the renin-angiotensin-aldosterone system in the control group. The medical community has a duty to closely monitor whether these questions will be raised.

And if the LCZ696 is released as an innovation in the treatment of heart failure, it is up to us, the cardiologists, to react with scientific maturity regarding the decision to whether or not use this treatment in our patients. Therefore, perhaps we will be preserving the countless individuals with heart failure from a pseudo-scientific conduct.
